# Assessing Income-Related Inequality on Health Service Utilization among Chinese Rural Migrant Workers with New Co-Operative Medical Scheme: A Multilevel Approach

**DOI:** 10.3390/ijerph182010851

**Published:** 2021-10-15

**Authors:** Dan Li, Shaoguo Zhai, Jian Zhang, Jinjuan Yang, Xiao Wang

**Affiliations:** 1 School of Public Management, Northwest University, Xi’an 710127, China; dandanli@nwu.edu.cn; 2 School of Public Policy and Administration, Xi’an Jiaotong University, Xi’an 710049, China; zhjian.2004@stu.xjtu.edu.cn; 3 Health Science Center, School of Public Health, Xi’an Jiaotong University, Xi’an 710061, China; yangjinjuan@stu.xjtu.edu.cn; 4 International Business School Suzhou, Xi’an Jiaotong-Liverpool University, Suzhou 215123, China; xiao.wang@xjtlu.edu.cn

**Keywords:** income-related inequality, health service utilization, Chinese rural migrant workers, New Co-operative Medical Scheme, multilevel approach

## Abstract

*Background:* Eliminating inequality in health service utilization is an explicit goal of China’s health system. Rural migrant workers with New Rural Cooperative Medical Insurance (NCMS) still face the dilemma of limited health service; however, there is a lack of analysis or measurement on the income-related inequality of health service utilization. *Method:* The nationally representative data of the China Labor-Force Dynamic Survey in 2016 were used for analysis. Multilevel regressions were used to obtain robust estimates and to account for various covariates associated with health service utilization of rural migrant workers with NCMS. The concentration index and its decomposition method were applied to quantify the income-related inequality of health service utilization of rural migrant workers. *Result:* The multilevel model analysis indicated that influencing factors of health service utilization were diversified, including gender, city service quality index, type of industry, the per capita annual income, marital status, health self-assessment, the community health index and the number of friends. The concentration indices of the total cost of inpatient and OOP cost of inpatient were 0.102 (95%CI: 0.031, 0.149), and the CI of OOP cost of inpatient was 0.094 (95%CI: 0.007, 0.119), respectively. The horizontal inequality indices of the total cost of inpatient and OOP cost of inpatient were 0.051 and 0.009, respectively. *Conclusion:* Our study presented a unique opportunity to examine the potential influence factors of health service utilization of rural migrant workers with NCMS, and highlighted that unequal health service utilization is evident among rural migrant workers with NCMS. This study provides important corroborative evidence to take full account of the contribution of each determinant to the inequality and health service needs among rural migrant workers with NCMS, in order to improve the basic medical insurance and social security systems—particularly for some marginal groups in China.

## 1. Background

With China’s reform and opening up, internal migration has been a phenomenon, especially after active measures channeling the transfer of rural labor after the 16th National Congress of the Communist Party of China. In China, the primary components of internal migrants are Chinese rural migrant workers, who always have been called “nongmingong”. As important drivers of social and economic development in China, Chinese rural migrant workers have made major achievements in the goal of building a moderately prosperous society in China. Rural migrant workers that face the cumulative effect of intensive work and low socioeconomic status might suffer from an exacerbation of their health status. A growing body of studies [1,2] has confirmed that Chinese rural migrant workers were more susceptible to poor health outcomes.

However, the Chinese household registration system (Hukou) required that each person born in China should be classified as either rural Hukou, or urban Hukou in a given location, leading to the dual characters of Hukou. On one hand, workers with different Hukou had different access to government-provided public health services and welfare programs in the urban areas. Migrants with rural and non-local Hukou working in cities had little access to welfare programs. On the other hand, there exists labor market discrimination against rural Hukou holders in cities, especially in the urban high-wage sector, such as state-owned enterprises [3]. Due to the dual characters of Hukou, the migration of rural migrant workers has resulted in problems caused by marginality in social status, unfair treatment and difficult integration of the new generation, which has brought great challenges to their health service utilization.

Major headway was made for basic health service utilization in China, especially the policy of New Rural Cooperative Medical Insurance (NCMS) declared in 2003, after the old cooperative medical scheme collapsed following economic reforms in the late 1970s. NCMS has offered comprehensive insurance coverage and fully subsidized insurance premiums to rural “Hukou” residents in China, and it has achieved remarkable achievements in reducing the obstruction to accessing services. However, in reality, not every rural migrant worker with NCMS can fully get an effective guarantee in NCMS as desired. The financial coordination and mutual aid of NCMS is carried out by the county or district as a management unit, leading NCMS with the characteristics of strong separability. For the most part, rural migrant workers take out NCMS in their hometowns. It is nearly impossible to transfer their NCMS between different cities and provinces in China because of different policies on NCMS declared by different places. Thus, they face the problem of off-site medical treatment of NCMS. Lower health insurance coverage in off-site treatment and complicated reimbursement procedures have increased the barriers to health service utilization, which could result in rural migrant workers with NCMS having to return to their hometown to reimburse their medical expenses, they even have to return to their hometown for medical treatment. What is worse is many rural migrant workers with NCMS are not clear about the policies on off-site medical treatment of NCMS. Integrating NCMS and the urban residents’ basic medical insurance system (URBMI) into the urban and rural residents’ basic medical insurance system (URRBMI) has been carried out since January 2016. However, to date, NCMS is still the major medical insurance for rural migrant workers. Therefore, an in-depth study on the NCMS would provide evidence to guide rural residents with NCMS or URBMI to seek medical treatment.

With the goal of reducing inequality in the world, the equality of health service utilization is highly ranked in the policy agenda. Many researchers [3,4] have paid special attention to the inequality of health service utilization of the floating population, the elderly, pregnant woman, etc. However, these conclusions may not be completely applicable to the analysis of rural migrant workers in China. Due to their Hukou, strong job mobility and high work intensity, rural migrant workers may be particularly vulnerable to equal opportunity in health service utilization. Literature abounds in the income-related health inequality of rural migrant workers. For example, Hong et al. [5] analyzed the health inequality of middle-aged and elderly rural migrant workers in China and found that health inequality significantly favored higher income groups.

They believed that better economic conditions could decrease their health inequality because high income meant that the long working hours and strong intensity of work for rural migrant workers, leading to greater health risks. However, only several pieces of literature [6] have drawn attention to the health inequality decomposition of rural migrant workers in China. The inequality of health service utilization of rural migrant workers in China has been a real problem in the process of social development. Unfortunately, there is a lack of knowledge about how to decompose the measured inequality of health service utilization of rural migrant workers into determinants.

As the improvement of income and living standards of Chinese rural migrant workers progresses, more equal opportunities are constantly required by Chinese rural migrant workers. However, their health service needs are often neglected and ignored. In line with our previous study [7], this study still augured that if we did not fully consider the need for health service utilization of rural migrant workers with NCMS, we would overestimate or underestimate the true inequality of their health service utilization. In our study, the concentration index decomposition method is used to disentangle inequality of the health service utilization of rural migrant workers with NCMS, which is conducive to an objective and comprehensive understanding of the health service utilization of rural migrant workers with NCMS.

Andersen’s model takes into account both societal and individual determinants from the perspective of systematic analysis aimed at understanding determinants of health services utilization, with three building blocks included predisposing, enabling and need variables [8]. Lee et al. [9] argued that the Andersen model provided the most appropriate analytical framework to explore the health service utilization of migrants and vulnerable groups. Related studies [10] demonstrated the effects of the explanatory variables of rural migrants workers using the original version of the Andersen model and found that that the current health delivery system is not conducive for rural migrants to seek appropriate health services. Chinese scholars [11] have expounded the index system of the Andersen Model (2013 Version) from a theoretical perspective since 2018, and the framework was composed of five dimensions: individual characteristics, health behavior, health outcome and contextual characteristics. The health service utilization of rural migrant workers is determined by the Chinese socio-cultural context, from the most intimate to the broadest socioeconomic factors, such as the cities and communities in which they live and the policies of a society (including NCMS). However, there is very little empirical study applying the Andersen Model (2013 Version) in the Chinese socio-cultural context, as the result of a lack of empirical research on the health service utilization of rural migrant workers.

Although there is an increasing number of evidence [12,13] that has demonstrated the status of the health service utilization of rural migrant workers and its associated factors, there are substantial knowledge gaps regarding rural migrant workers with NCMS in China. What’s more, the nationally representative data we used showed a hierarchical structure. Generally speaking, the heterogeneity among rural migrant workers with NCMS living in the same community or city is smaller than that among those living in different communities or cities, which would violate the classical assumption of the single-level regression model. If only crude single-level regression models are used, the independent variables are assumed to be independent and equal in the probability. Multilevel models became a widely affirmed approach because they explicitly recognize the hierarchical structure of the data (in our study, rural migrant workers with NCMS clustered within communities or cities) and overcome the inadequate assumption of independence among units belonging to the same communities or cities. Thus, we adopted a multilevel regression approach to fully meet our needs.

Assessing the inequality and to what extent the inequality existed in the health service of the vulnerable groups is highly relevant in today’s sociopolitical climate, given the targets set in the 14th five-year plan in China. In our study, we used a three-level design where rural migrant workers are nested within communities and cities to offer an explanation for the potential influence factors of health service utilization of rural migrant workers with NCMS. In particular, we examined the inequality of health service utilization of rural migrant workers with NCMS and decomposing the measured inequality into determinants. Our result would be referential for the positive policy exploration for rural migrant workers in China.

## 2. Methods

### 2.1. Data

The data was collected from China Labor-Force Dynamic Survey in 2016 (CLDS 2016), the data of Urban Statistics Yearbook and Statistics Bulletin. A total of 21,086 respondents aged 15~64 were recruited in CLDS 2016. We obtained the detailed demographic, health, economic and health service utilization from CLDS 2016. We thus identified rural migrant workers participating in NCMS aged 15–64. Informed by the Center for Health Statistics and Information, rural migrant workers are defined as those who are rural labor forces, engaged in non-agricultural industries or have worked outside the country for 6 months or more [14]. We included rural migrant workers who only participated in NCMS, not participating in other basic medical insurance. Finally, the sample was reduced to 3322 respondents after data cleaning (i.e., excluding ages 15–64, illogical answers, more than 15% data missing for one variable). Missing data occurred in less than 5% of responses to most questions in our study. Therefore, we replaced the missing values with the mean, if the missing data were numerical, and we replaced the missing values with mode if the missing data were categorical.

### 2.2. Measurements

The following four dependent variables were included and presented by: 

Question 1: how much did you spend on the two-week visit to the clinic in total (including reimbursable or reimbursable)? 

Question 2: how much did you pay out of pocket on the two-week visit to the clinic (including reimbursable or reimbursable)?

Question 3: how much did you spend on your hospitalization since July 2015 in total (including reimbursable or reimbursable)? 

Question 4: how much did you pay out of pocket on hospitalization since July 2015 (including reimbursable or reimbursable)?

### 2.3. Predictor

The Andersen Model (2013 Version) provides a reasonable and reliable theoretical framework to take a number of factors into account, including social-political setting, the existing health infrastructure and self-reported measures. In our study, we used the Andersen Model (2013 Version) to study the health services used by Chinese migrant workers. The variables included:

(1) Individual characteristics: age group (age 50–60 = 0, 61 and above = 1), gender (male = 0, female = 1), living arrangement (live with spouse = 0, live without spouse = 1), educational attainment (below primary school = 0, primary school = 1, middle school and above = 2), technical certificate (yes = 0, no = 1), type of industry (professional technician/clerical staff = 0, service stuff = 1, manufacturing and construction = 2, freelancer = 3), type of unit (party/government/state-owned/collective enterprises and institutions = 0, private/foreign/joint venture = 1, self-employed and freelance = 2), migration distance (in the county/district = 0, across the county/district = 1), working hour (moderate labor = 0, excessive labor = 1), income quintiles (the poorest = 0, the poorer = 1, the middle = 2, the richer = 3, the richest = 4), injury insurance (yes = 0, no = 1), number of friends (less than or equal to 5 = 1, 6~10 = 2, more than or equal to 11 = 3), self-assessed of health status (SAH) (good = 0, fair = 1, poor = 2). In lines with previous studies [15,16], excessive labor was defined as working 50 or more hours per week, and moderate labor was defined as working less than 50 h per week.

(2) Health behavior: smoking (yes = 0, no = 1), alcohol use (yes = 0, no = 1), regular exercise every month (yes = 0, no = 1).

(3) Health outcome: sense of fairness (unhappy = 0, fair = 1, happy = 2).

(4) Contextual characteristic: the proportion of ethnic minorities, per capita in the community, service quality index of the community, region (east = 0, central = 1, west = 2), city-level reflecting the political rule and the policy-oriented factors in China (sub-provincial city and above = 0, below sub-provincial city = 1), service quality index of the city, health index of the community, the number of medical institutions per 10,000 people in the community, the number of medical institutions per 10,000 people in the city, the number of beds per 10,000 people in the city, and the number of doctors per 10,000 people in the city. However, the Chinese government or other entities have not announced official data related to rural areas. The empirical analysis encounters data constraints. Our study insisted that the urban service quality index can reflect the service quality of different cities, and then it would, in turn, reflect the rural service quality index to a certain extent. Although some hidden bias may remain, we think that the urban service quality index in the same local city provided a better alternative to compare the service quality of different cities than other indicators before the national official publication of the rural Statistical Year. In our study, we adopted the exploratory factor analysis method [17] to scientifically construct three indices: a service quality index of the city, a service quality index of the community and a health index of the community, to avoid the collinearity among the variables in the comprehensive evaluation of cities and communities. In order to avoid the error caused by the order of magnitude or dimension of the original data, the original data was standardized, the average number was 0, the variance was 1, and then the linear distribution of each factor can be seen by adding the coefficients. The factors were weighted to sum by the weight of variance contribution, and then the index was evaluated by the comprehensive score. The KMO value of the three indices was greater than 0.6, and the *p*-value of the Bartlett test of the three indexes was less than 0.001. Both results indicate that the three indexes were suitable for the exploratory factor analysis method [18].

### 2.4. Multilevel Regression Model

Analyzing variables from different levels without taking into account the hierarchical structure of “city-community-rural migrant workers with NCMS” would lead to misleading estimation and interpretation because of the problem of heteroscedasticity. To overcome such problems, a multilevel regression model has been developed to satisfy the assumption of independence and heteroscedasticity, which would avoid the error terms and the “mean square error” of the city or the community, especially when the outcome variable is dependent on the clustering. The Intra-Class Correlation Coefficient (ICC) is the ratio of the between-group variance to the total variance; the calculation formula of ICC could be specified as:

(1) $$\mathrm{ICC}=\frac{\sigma_{u0}^{2}}{\sigma_{u0}^{2}+\sigma_{e0}^{2}}$$

The data have a three-level hierarchical structure with rural migrant workers with NCMS at level 1, nested within the community at level 2, and nested within the city at level 3. $\sigma_{u0}^{2}$, which presents the between-group variance in the same level, $\sigma_{e0}^{2}$ presents rural migrant workers with NCMS variance in different levels. When the ICC is closer to 0, indicating it to be more independent, multilevel models between and within cluster components of the first-level covariatesare distinguished. Then perform significantly better than both multilevel models where the two-effects are set to be equal to the fixed-effect model. When ICC is significantly larger than 0.059, multilevel regression models would be adopted, and multilevel regression models allow explanatory variables to be entered into the model so that the causes of response inconsistency or differential test functioning can be investigated [19]. 

The multilevel model was step wise. In the first step, the intercept only or the empty model was fitted to check the hierarchical structure of the data. ICC can be judged whether to use a multilevel model or not. In the next step, the random variables were added, including variables representing the fixed-effect to add to the high-level explanatory variables. The intercept and fixed slope model, usually called the random intercept model, was fitted, and finally, the random intercept and random slope (random slope model) were fitted. The random slope in low level can be tested to adjust for the effects of the level of rural migrant workers with NCMS.

The three-level regression model is expressed as follows:

(2) $$\mathrm{logit}\left(\frac{p_{\mathrm{ijk}}}{1-p_{\mathrm{ijk}}}\right)={\beta x}_{\mathrm{ijk}}+{\gamma \omega }_{\mathrm{jk}}+{\eta z}_{k}+\mu_{\mathrm{jk}}+\nu_{k}$$

In this 3-level regression model, i, j, k, represent level 3—city, level 2—community, and level 1—rural migrant workers with NCMS, respectively. $x_{\mathrm{ijk}}$, $\omega_{\mathrm{jk}}$ and $z_{k}$ are the regression coefficients corresponding to the explanatory variables, respectively. β, γ and η represent the estimated value of the regression coefficient of the explanatory variable at each level. $\mu_{\mathrm{jk}}{\;\mathrm{and}\;\nu }_{k}$ represent the errors of level 2-community and level 3-city, respectively. All three random effects are assumed to be independent and normally distributed. The three-level linear regression model can be written as follows, splitting up into three models:

(3) $$y_{\mathrm{ijk}}={\beta x}_{\mathrm{ijk}}+{\gamma \omega }_{\mathrm{jk}}+{\eta z}_{k}+\mu_{\mathrm{jk}}+\nu_{k}$$

i, j, k represent a level-3 city, level 2-community, and level-1 rural migrant workers with NCMS. $x_{\mathrm{ijk}}$,${\;\omega }_{\mathrm{jk}}$, $z_{k}$ representing the explanatory variables of three levels. β, γ, η represent the estimated value of the regression coefficient of the explanatory variable at each level. $\mu_{\mathrm{jk}},\nu_{k}$ represent the residuals of a level 2 community and level 3 city, respectively.

### 2.5. Concentration Index and Decomposition

The Gini index and Concentration index (CI) were widely used to quantify inequality as the function of differences between shares of some health outcomes compared with shares of the population. CI expressed the degree of socioeconomic-related inequality in a health variable (e.g., health status, health service utilization) [20,21]. What’s more, CI is a scientific and effective method for measuring the degree of socioeconomic-related inequality in health service utilization in one variable related to the ranking of another variable (e.g., income), because it does not only overcome the defects of the Gini index which only takes positive variables but also it can be decomposed proportionally into contributions of different inequality of socio-economic factors [21,22]. CI was adopted to analyze and compare the health service utilization of rural migrant workers with different economic statuses in order to determine whether their health services utilization was equal. CI lies between −1 and +1, where 0 indicates no income-related inequality of health service utilization of rural migrant workers with NCMS, a positive (negative) score when there is income-related inequality of health service utilization of rural migrant workers with NCMS favoring the rich (poor) [23,24]. Equation (1) can be calculated the CI:

(4) $$\mathrm{CI}=\frac{2}{\mu }\mathrm{cov}\left(y_{i},R_{i}\right)$$

where CI indicates the concentration index of health service utilization of rural migrant workers with NCMS, indicates the health service utilization, μ is the mean value of health service utilization and is the ranking of the economic status of rural migrant workers with NCMS.

The concentration index of each factor was decomposed into the contribution of each factor to the income-related inequality in health service utilization while controlling for other determinants [25,26]. The formula of the model was:

(5) $$y=\alpha^{m}+\sum_{j}\beta_{j}^{m}x_{j}+\varepsilon$$

where *y* is the health service utilization indicator. We computed the marginal effect of income for the computation. $\beta_{j}^{m}$ is partial effects (i.e., dy/dxj) of each variable and evaluated at sample means; $\alpha^{m}$ is the constant term in the regression equation, ε is the error term. Calculating the CI of Equation (5) and the decomposition of the CI is as follows:

(6) CI=∑j(βjmxj¯μ)Cj+GCε

where *μ* is the mean of the dependent variable; *C_j_* is the concentration index of *x_j_*; is the means of *x_j_*; is the elasticity of in health service utilization and *G* is the elasticity of in health service utilization of rural migrant workers with NCMS. When a variable is the only impact factor, a positive contribution rate indicates that the variable increased the inequality of health service utilization of rural migrant workers with NCMS and vice versa.

CI of health service utilization of rural migrant workers with NCMS was equal to the weighted sum of the CI of the “need” variable and other control variables (the residual term was not considered). The horizontal inequality index was calculated by subtracting the needs of health service utilization from the CI of health service utilization of rural migrant workers with NCMS. The formula is as follows:

(7) $$\mathrm{HI}=\mathrm{CI}-\underset{j}{\sum }(\beta_{j}^{m}x_{ji}/\mu )C_{j}$$

$\beta_{j}^{m}$ presents the partial regression coefficient of the variable of health service need. The “need” variable in our study included age, gender and SAH. *x_j_* and *c_j_* presents the mean and the CI of health service need variable. *μ* presents the mean of outcome variable.

All analyses were undertaken using Stata statistical software version 15.0 (StataCorp LP., College Station, TX, USA). The probability, *p*-value ≤ 0.05 was considered to indicate statistical significance.

## 3. Result

Table 1 reported summary statistics of respondents. There is a great heterogeneity within rural migrant workers with NCMS. 

As shown in Table 2, the mean value of the total cost of outpatient was 576.93 yuan, and the median was 300.00 yuan. The mean value of the total cost of inpatient was 6719.74 yuan, and the median was 5000.00 yuan. The mean value of OOP cost of outpatient was 447.16 yuan, and the medianof OOP cost of outpatient was 200.00 yuan. The mean value of OOP cost of inpatients was 4269.29 yuan, and the median of OOP cost of inpatients was 3000.00 yuan. 

Taking community as level 2 and rural migrant workers with NCMS as level 1, the two-level model without any explanatory variables was fitted, and the fixed-scale parameter was 1. Table 3 presented that the variance of community level of the expenses of two-week outpatient service and inpatient service were 0.647, 0.556, 3.46 × 10^−16^ and 2.79 × 10^−17^, respectively. Then, the ICC of level-community can be calculated to be 0.127, 0.095, 2.50 × 10^−16^ and 8.47 × 10^−18^, respectively.

Table 4 presented the estimations of the three-level (level-rural migrant workers with NCMS, level-community and level-city) multilevel regression models. The variance of community level of the expenses of two-week outpatient service and inpatient service was 0.459, 0.430, 4.11 × 10^−15^ and 0.430, respectively. Then, the ICC of level-city and level-community of the total cost of outpatient can be calculated to be 0.090 and 0.119, respectively. The ICC of level-city and level-community of OOP cost of outpatient can be calculated to be 0.073 and 0.088, respectively. The ICC of level-city and level-community of the total cost of inpatient can be calculated to be 2.92 × 10^−15^ and 2.95 × 10^−15^, respectively. The ICC of level-city and level-community of OOP cost of inpatient can be calculated to be 2.92 × 10^−15^ and 2.92 × 10^−15^, respectively. The ICC of the total cost of outpatient and OOP cost of outpatient resulted in large bias in the model, but the ICC of the total cost of inpatient and OOP cost of inpatient resulted in minor bias in the model. Therefore, it is only necessary to use the multilevel model to analyze the total cost of outpatient and OOP cost of outpatient among rural migrant workers with NCMS, and no multilevel analysis is required of the total cost of inpatient and OOP cost of inpatient.

Table 5 presented the adjusted associations between health service utilization of rural migrant workers with NCMS and its determinants. The significant influencing factors of the total cost of two-week outpatient migrant workers included gender and city service quality index. The total cost of female rural migrant workers was lower than that of men (Coef. = −0.867, *p* < 0.05). There is a positive relationship between the city service quality index and the total cost of the two-week outpatients (Coef. = 0.904, *p* < 0.05). The significant influencing factors of the OOP cost of the two-week outpatient migrant workers included the type of industry and the per capita annual income. The OOP cost of two weeks working in collective enterprises and institutions was significantly lower than those in Party/government/state-owned enterprises and institutions (Coef. = −1.760, *p* < 0.05). There is a positive relationship between the per capita annual income and the OOP cost of two-week outpatients (Coef. = 1.04 × 10^−10^, *p* < 0.05). The significant influencing factors of the total cost of inpatient of migrant workers included marital status, health self-assessment and the community health index. The total cost of inpatient of rural migrant workers with spouses was significantly higher than rural migrant workers without spouses (Coef. = −0.545, *p* < 0.05). The total cost of inpatient of rural migrant workers with poor self-assessment was significantly higher than that of migrant workers with better self-assessment (Coef. = 0.513, *p* < 0.05). There is a positive relationship between the community health index and the total cost of two-week outpatients (Coef. = 0.541, *p* < 0.05). The OOP cost of inpatient of participating migrant workers with the number of Friends >= 11 was significantly lower than those with the number <= 5 (Coef. = −1.015, *p* < 0.05).

Table 6 showed the total cost and OOP of two-week outpatients and inpatients among rural migrant workers with NCMS in different economic statuses in China. The total cost of two-week outpatients among rural migrant workers with NCMS in five economic groups was 462.40 Yuan, 731.86 Yuan, 427.86 Yuan, 690.04 Yuan and 572.48 Yuan, respectively. The OOP cost of two-week outpatients among rural migrant workers with NCMS in five economic groups was 414.63 Yuan, 580.38 Yuan, 325.18 Yuan, 497.37 Yuan and 418.21 Yuan, respectively. The total cost of inpatients among rural migrant workers with NCMS in five economic groups was 6365.16 Yuan, 4353.10 Yuan, 7039.63 Yuan, 7846.43 Yuan and 8346.19 Yuan, respectively. The OOP cost of inpatient among rural migrant workers with NCMS in five economic groups was 4217.92 Yuan, 2641.95 Yuan, 4540.65 Yuan, 4778.55 Yuan and 5160.43 Yuan, respectively.

The inequalities of two-week outpatient costs and inpatient costs among rural migrant workers with NCMS were measured by CI (Table 7). The CI of the total cost of outpatients was 0.009 (95% confidence interval: −0.005, 0.133), and the CI of OOP cost of outpatients was −0.026 (95% confidence interval: −0.402, 0.171). The CI of the total cost of inpatients was 0.102 (95%CI: 0.031, 0.149), and the CI of OOP cost of inpatients was 0.094 (95%CI: 0.007, 0.119). The significantly positive value of the CIs indicated strong pro-rich inequalities, that is, rich rural migrant workers with NCMS had more medical costs than the poor.

Inequalities of the total cost and OOP of two-week outpatient and inpatient can be further explained by decomposing the CIs into the determining components. The decomposition of the concentration index of each health service utilization indicator was shown in Table 8. Only CIs of the total cost of inpatient and OOP cost of inpatient was statistically significant, then we showed the decomposition of the concentration index of the total cost of inpatients and OOP cost of inpatients. The factors that contributed to the inequality of total cost of inpatients were as follows: women (121.41%), self-employed and freelance (−74.35%), collective enterprises and institutions (64.51%), poorer group (−48.23%), richer group (57.27%), no smoking (−53.55%), number of medical institutions for 10,000 people in the city (36.35%), fair SAH (−35.88%) and having no injury insurance (−32.24%). The factors that contributed more to the inequality of the OOP cost of inpatients were women (149.47%), no smoking (−86.69%), richer group (85.02%), middle group (44.89%), Service staff (−41.93%), poorer group (−40.40%), number of medical institutions for 10,000 people in the city (40.56%) and having no injury insurance (−32.55%).

As Table 9 showed, the contribution rates of need to the inequalities of the total cost and OOP cost of two-week outpatient were 15.84% and −38.22%, respectively. HIs of the total cost and OOP cost of two-week outpatient were 0.010 and −0.036, respectively. The contribution rates of need to the inequalities of the total cost and OOP cost of inpatient were 91.71% and 123.64%, respectively. HIs of the total cost and OOP cost were 0.051 and 0.009, respectively. It showed that the need for health service utilization decreased the inequality of the total cost and OOP cost of two-week outpatient of richer rural migrant workers with NCMS, but it increased the inequality of the total cost and OOP cost of inpatient of richer rural migrant workers with NCMS.

## 4. Discussion

This novel study examined the inequality of outpatient and inpatient utilization of rural migrant workers with NCMS in China, the country with the largest scale of internal migration. This study analyzed determinants of health service utilization among rural migrant workers with NCMS using the multilevel modeling technique with an application to hierarchical nationally representative data from CLDS 2016. To our knowledge, this study was the first to investigate whether and to what extent inadequate access to health service utilization among rural migrant workers with NCMS using a large-scale representative sample. Then, the existence of pro-rich inequalities of inpatient service was evident by the results from the horizontal inequality analysis.

The stratified sampling conducted by CLDS 2016 inevitably resulted in a hierarchical structure of data. Compared with the single-level model, multilevel models can decompose the random errors into multiple levels and construct the error structure which is suitable to the hierarchy data. It provided an approach to solve problems caused by single-level models to a great extent. A multilevel approach can not only estimate the parameters of individual-level variables more accurately but also accurately and comprehensively explain different levels of feature variables. It should be pointed out that the data structure in this study actually showed four levels of “region-city-community-rural migrant workers with NCMS”. However, if the level of the region is added to the analysis, the parameter estimation of the four-level model and the overall fitting effect of the model would be worse than those of the three-level model. Therefore, our study did not regard the regional level as the fourth level. Finally, a three-level model of influencing factors on health service utilization of participating migrant workers was established, which was based on “city-community-rural migrant workers with NCMS”.

Our results showed that men had higher expenses on the two-week outpatient utilization compared to women. That’s mainly related to the nature of the work of rural migrant workers, and male rural migrant workers were more engaged in high-intensity physical labor, which increased the probability of injury, causing the higher medical cost. But the gender is not statistically significant in the inpatient. It may be explained that, besides the nature of their work, pregnancy is an important point of entry to health utilization services, and many female rural migrant workers in China are of reproductive age. The city service quality index, community health index and the per capita annual income increased the medical costs of rural migrant workers with NCMS. Therefore, we should pay attention to improving the service quality in communities and cities where rural migrant workers lived in. The OOP cost of two-weeks of rural migrant workers with NCMS working in collective enterprises and institutions were significantly lower than those in Party/government/state-owned enterprises and institutions, indicating the importance of stable work and stable income. Another point is that whatever type of industry rural migrant workers are engaged in, they would get the opportunity for sick leave, which is beneficial to their health by facilitating timely and quality medical care to screen for and treat diseases in the early stages. It is commonly accepted that the costs of a two-week visit and hospitalization of rural migrant workers with fair SAH and poor SAH were higher than those with good SAH. In line with our previous studies [1,8], our results fully demonstrated the importance of good SAH to the two-week visit and hospitalization of rural migrant workers with NCMS. Substantial literature argued the effect of marriage on health. On one hand, married individuals may be self-selected based on health-related characteristics, attitudes toward health, or behavioral factors, due to “marriage selection” [27]. On the other hand, literature in support of “marriage protection” implied that a protective role of spouses (especially women) providing physical and emotional support, resulted in better health [28]. Our findings showed that the total cost of inpatient of rural migrant workers with spouses was lower, and it supported the assertion that marital status is an important predictor of their health to a certain extent.

In terms of total cost and the OOP costs of the two-week outpatient, both CIs were not statistically significant. For inpatients, the total cost and the OOP cost showed a statistically significant CI of 0.102 (95% CI: 0.031, 0.149) and 0.094 (95% CI: 0.007, 0.119). Our results, as well as previous studies [8], demonstrated that total costs and OOP costs of inpatients are unequally distributed among the income spectrum. Our results consistently revealed that the wealthy rural migrant workers had greater economic advantages in health service utilization than their poor counterparts. Our study cannot analyze rural migrant workers who prefer self-care methods, such as, going to pharmacies to buy drugs, because they would not likely sacrifice their working hours to go to the hospital. Rural migrant workers suffer from unnoticed high health risks that can wear off their health risk awareness and make them vulnerable to long-term health problems [29,30]. What’s worse, many rural migrant workers do not pay attention to the physical examination. However, related studies [11] showed that medically unexplained symptoms would be more likely to indicate an important burden to the healthcare system, and they even account for approximately a quarter to a half of all patient visits in primary care settings in developed countries.

The contributors of the inequality of total costs and OOP costs of inpatients were gender, type of unit, economic level, having injury insurance, SAH, smoking and the number of medical institutions for 10,000 people in the community. Consistent with previous studies [31], women with relatively lower economic status were more vulnerable to diseases, and women conversely improved the pro-rich inequality in total costs and OOP costs of inpatient among rural migrant workers with higher economic levels. Our findings raised the possibility that the type of unit may influence health status not only through the support and protection that employment offers but also through a more efficient pattern of health service utilization.

Under the guarantee of the reimbursement policy of NSMS for inpatient, rural migrant workers with NCMS with better income levels would choose medical quality treatment or superior treatment environments and therefore, the total costs and the OOP costs tended to be higher than those with low income. The inequality of health services should be taken seriously, especially for disadvantaged rural migrant workers. Our findings also have potentially important implications make income distribution more reasonable and orderly within rural migrant workers with NCMS in China. Since the type of industry had a significant impact on health service utilization, rural migrant workers with NCMS with a steady income would reduce the wealth-favored inequality in inpatient service utilization of rural migrant workers with NCMS. Insurance against injury at work would reduce the inequality of the high total costs and OOP costs of rural migrant workers with NCMS with lower economic levels and therefore, we should pay attention to insurance against injury at work. The number of medical institutions in the community increased the inequality of total costs and OOP costs of inpatient among rural migrant workers with better economic status. An important implication of this paper is that the government should pay attention to the inadequate access to health services in the community and provide better health services for rural migrant workers with NCMS. When it comes to inequality of health service utilization, the internal passport system may obstruct equity appearing in other countries, for example, Australia, Japan [32]. The Hukou system of permanent registration favors urban residents and discriminates against rural residents in resource allocation, consequently widening inequality in China. Furthermore, our findings had implications for the improvement of health care delivery systems, which exacerbate the burdens on health service management systems.

As we mentioned, the inequality of health service utilization caused by the health service need is reasonable, while the inequality caused by non-health service demand can be understood as the horizontal inequality, which was used to measure unequal access to health services within a cluster. After controlling the effect of the health service need variables (age, gender, SAH) on the inequality of health service utilization, the horizontal inequality index of the total cost of inpatient and the OOP cost of inpatient were 0.051 and 0.009, respectively, indicating the presence of a pro-rich inequality among health service utilization. Furthermore, the horizontal inequality was higher in the total cost of inpatients than in the OOP cost of inpatients. In fact, the income-related inequality of rural migrant workers with NCMS cannot be blamed on their “not working hard”, a greater understanding of true inequality have important implications for health care and social policy, which may hold the potential to optimize health system design. What’s more, adopting feasible strategies is an important part of the project’s “Health China2030”to build moderately prosperous goals by 2030. In order to translate our findings into relevant policy, it is important to understand the health service’s need for health service utilization.

Our findings should be interpreted in light of several limitations. First, the cross-sectional analysis of secondary data assessed an association but did not allow such causality to be determined. Although we have tried to control for the potential selection as rigorously as our data would allow through multilevel regression controls, it is possible that unobserved clinical characteristics actually mediate our findings. Further findings valuable for plausible causal inference should be concluded. Second, considering the availability of data, the database lacks information on the severity of diseases, type of treatment received, chronic disease status, disability-adjusted life years, etc.Third, medical expenses and consumption expenditure were based on the respondents’ self-reported information, which may be prone to memory biases due to the limited recall period. We did recognize that self-assessments may not reflect actual medical expenses. However, previous studies [33] suggested that self-reported health service utilization is highly aligned with actual health service utilization. Finally, the problem addressed in our study is generic on a global level after the pandemic of COVID-19. Our future studies will compare similar scenarios in one or two other APAC regions and try to make more comprehensive analyses to look into the post-COVID-19 trends.

## 5. Conclusions

In conclusion, our study presented valuable information on the inequities in the health service utilization and the health service need of rural migrant workers with NCMS in China. Our findings highlighted that substantial inequalities in health service utilization existed. Additionally, the need for pro-rich inequality in the inpatient was observed. Our findings underscore the importance of health service needs. Based on the contributing factors, our study identified opportunities on compensation policies and benefit packages to improve the equality for disadvantaged rural migrant workers with NCMS.

## Figures and Tables

**Table 1:** Statistics for the characteristics of respondents.

Variables	Number/Mean	Percentage (%)/SD
Individual characteristics		
Age group		
15~36 ^†^	1303	39.22
36~50	1199	36.09
50~64	820	24.68
Gender		
Men ^†^	1910	57.50
Women	1412	42.50
Living arrangement		
Live with spouse ^†^	500	15.05
Live without spouse	2822	84.95
Educational attainment		
Below primary school ^†^	923	27.78
Primary school	1619	48.74
Middle school and above	780	23.48
Technical certificate		
Yes ^†^	422	12.70
No	2900	87.30
Type of industry		
Professional technician/Clerical staff ^†^	248	7.47
Service stuff	1177	35.43
Manufacturing and construction	1041	31.34
Freelancer	856	25.77
Type of unit		
Party/government/state-owned ^†^	300	9.03
Collective enterprises and institutions	1327	39.95
Self-employed and freelance	1695	51.02
Working hours		
Moderate labor ^†^	1471	44.28
Excessive labor	1851	55.72
Place of work		
In the county/district ^†^	2721	81.91
Across the county/district	601	18.09
Income quintiles	664	19.99
Poorest ^†^	665	20.02
Poorer	664	19.99
Middle	665	20.02
Richer	664	19.99
Richest	664	19.99
Injury insurance		
Yes ^†^		
No	293	8.82
number of friends	3029	91.18
<=5 ^†^		
6~10	1904	57.31
>=11	811	24.41
SAH	607	18.27
Good ^†^		
Fair	2285	68.78
Poor	837	25.20
health behavior		
Smoke		
Yes ^†^	1192	35.88
No	2130	64.12
Alcohol use		
Yes ^†^	831	25.02
No	2491	74.98
Regular exercise every month		
Yes ^†^	818	24.62
No	2504	75.38
Health outcome		
Sense of happiness		
Unhappy ^†^		
Fair	215	6.47
Happy	1014	30.52
Contextual characteristic		
Proportion of ethnic minorities	1.000	0.006
Per capita in the community	1.000	2.02 × 10^−4^
Region		
East ^†^	2074	62.43
Middle	639	19.24
West	609	18.33
City level		
Sub-provincial city and above	570	17.16
Other	2752	82.84
Number of medical institutions for 10,000 people in the community	5.60	18.48
Number of medical institutions for 10,000 people in the city	2601.65	4597.18
Number of doctors for 10,000 people in the city	7.48	12.24
Number of beds for 10,000 people in the city	0.70	1.33
Health index of the community	54.34	19.24
Service quality index of the community	83.94	44.33
Service quality index of the city	−0.05	0.64
Intercept	0.07	0.24

Note: ^†^ Reference levels in the regressions; SD: standard deviation.

**Table 2:** Total cost and OOP cost of health service utilization.

	Two-Week Outpatient		Inpatient	
	M (SD)	Median (ID)	M (SD)	Median (ID)
Total cost (yuan)	576.93 (683.79)	300.00 (410.00)	6719.74 (5554.01)	5000.00 (7500.00)
OOP cost (yuan)	447.16 (506.71)	200.00 (355.45)	4269.29 (4499.64)	3000.00 (4000.95)

Note: M for mean value; SD for standard deviation; ID for inter-quartile distance.

**Table 3:** Two empty models of influencing factors of health service utilization.

Variables		Total Cost of Outpatient		OOP Cost of Outpatient		Total Cost of Inpatient		OOP Cost of Inpatient	
		Coef.	SE	Coef.	SE	Coef.	SE	Coef.	SE
Fixed effects									
	Intercept	5.768 ***	0.169	5.336 ***	0.178	8.788 ***	0.084	8.062 ***	0.129
Random effects									
	Community level variance	0.647	0.435	0.556	0.458	3.46 × 10^−16^	1.29 × 10^−15^	2.79 × 10^−17^	1.17 × 10^−16^
	Personal level parameter	1.000	0.000	1.000	0.000	1.000	0.000	1.000	0.000

Note: Estimates of random-effect parameters and residual variance parameters were reported as standard errors. Coef. for Coefficient; SE for standard error; *** *p* < 0.001.

**Table 4:** Three-level empty model of influencing factors of health service utilization.

Variables		Total Cost of Outpatient		OOP Cost of Outpatient		Total Cost of Inpatient		OOP Cost of Inpatient	
		Coef.	SE	Coef.	SE	Coef.	SE	Coef.	SE
Fixed effects									
	Intercept	5.839 ***	0.184	5.393 ***	0.193	8.773 ***	0.086	5.393 ***	0.193
Random effects									
	City level variance	0.459	0.422	0.430	0.397	4.11 × 10^−15^	4.19 × 10^−14^	0.430	0.397
	Community level variance	0.145	0.480	0.089	0.508	3.89 × 10^−17^	2.08 × 10^−16^	0.089	0.508
	Personal level parameter	1.000	0.000	1.000	0.000	1.000	0.000	1.000	0.000

Note: Estimates of random-effect parameters and residual variance parameters were reported as standard errors. Coef. for Coefficient; SE for standard error; *** *p* < 0.001.

**Table 5:** Association of independent variables and health service utilization costs.

Variables	Total Cost of Outpatient		OOP Cost of Outpatient		Total Cost of Outpatient		OOP Cost of Outpatient	
	Coef.	SE	Coef.	SE	Coef.	SE	Coef.	SE
Individual characteristics								
Age group								
15~36 ^†^								
36~50	−0.184	−0.433	−0.322	−0.462	0.277	0.264	0.662	0.432
50~64	0.415	−0.468	0.53	−0.499	0.366	0.280	0.387	0.458
Gender								
Men ^†^								
Women	−0.867 *	−0.42	−0.622	−0.447	−0.118	0.282	−0.307	0.461
Living arrangement								
Live with spouse ^†^								
Live without spouse	−0.36	−0.387	−0.242	−0.412	−0.545 *	0.294	−0.676	0.480
Educational attainment								
Below primary school ^†^								
Primary school	−0.081	−0.374	0.159	−0.398	0.171	0.235	−0.085	0.384
Middle school and above	0.178	−0.529	0.072	−0.564	0.138	0.306	0.419	0.501
Technical certificate								
Yes ^†^								
No	−0.056	−0.471	0.181	−0.502	−0.262	0.276	−0.299	0.452
Type of industry								
Professional technician/Clerical staff ^†^								
Service stuff	0.035	−0.635	0.844	−0.677	0.093	0.461	−0.532	0.753
Manufacturing and construction	0.607	−0.674	0.897	−0.719	−0.404	0.469	−0.912	0.766
Freelancer	0.43	−0.724	1.23	−0.771	−0.187	0.525	−0.816	0.858
Type of unit								
Party/government/state-owned ^†^								
Collective enterprises and institutions	−0.954	−0.612	−1.760**	−0.652	−0.350	0.398	−0.329	0.651
Self-employed and freelance	−0.457	−0.655	−1.179	−0.698	−0.606	0.4112	−0.150	0.672
Working hours								
Moderate labor ^†^								
Excessive labor	−0.044	−0.312	−0.468	−0.333	0.169	0.1841	−0.022	0.300
Place of work								
In the county/district ^†^								
Across the county/district	0.085	−0.479	−0.292	−0.51	−0.045	0.254	0.318	0.520
Income quintiles								
Poorest ^†^								
Poorer	0.369	−0.431	0.361	−0.46	0.040	0.264	0.079	0.432
Middle	0.527	−0.439	0.795	−0.468	0.219	0.292	0.494	0.477
Richer	0.451	−0.504	0.55	−0.537	0.326	0.306	−0.097	0.501
Richest	−0.643	−0.587	−0.948	−0.626	0.116	0.325	−0.229	0.531
Injury insurance								
Yes ^†^								
No	−0.154	−0.514	0.27	−0.547	0.069	0.385	0.839	0.629
number of friends								
<=5 ^†^								
6~10	0.003	−0.359	0.077	−0.383	0.046	0.240	0.258	0.392
>=11	0.283	−0.533	−0.01	−0.568	−0.378	0.248	−1.015 *	0.405
SAH								
Good ^†^								
Fair	−0.203	−0.39	−0.054	−0.416	−0.064	0.216	0.008	0.352
Poor	0.523	−0.478	0.65	−0.509	0.513 *	0.248	−0.012	0.406
health behavior								
Smoke								
Yes ^†^								
No	0.02	−0.42	0.378	−0.447	−0.083	0.272	0.079	0.444
Alcohol use								
Yes ^†^								
No	0.482	−0.431	0.183	−0.459	−0.177	0.247	−0.029	0.404
Regular exercise every month								
Yes ^†^								
No	−0.391	−0.346	−0.316	−0.369	−0.241	0.198	−0.002	0.324
Health outcome								
Sense of happiness								
Unhappy ^†^								
Fair	0.158	−0.459	0.585	−0.489	0.244	0.357	0.462	0.584
Happy	0.673	−0.465	0.854	−0.495	0.515	0.331	0.682	0.542
Contextual characteristic								
Proportion of ethnic minorities	0.014	−0.007	0.011	−0.008	−0.001	0.004	−0.004	0.007
Per capita in the community	1.00 × 10^−5^	1.21 × 10^−6^	1.04 × 10^−10^ *	1.23 × 10^−7^	1.83 × 10^−5^	1.01 × 10^−5^	2.06 × 10^−5^	1.85 × 10^−5^
Region								
East ^†^								
Middle	−0.532	−0.547	−0.341	−0.583	−0.2129	0.273	−0.223	0.446
West	−0.135	−0.618	−0.213	−0.659	−0.1281	0.305	0.368	0.498
City level								
Sub-provincial city and above								
Other	−0.575	−0.542	−0.892	−0.578	0.183	0.276	−0.201	0.451
Number of medical institutions for 10,000 people in the community	−94.052	−179.788	−119.904	−191.645	−0.014	0.010	−0.018	0.016
Number of medical institutions for 10,000 people in the city	−0.111	−0.185	0.042	−0.197	−0.072	0.089	−0.098	0.146
Number of doctors for 10,000 people in the city	−0.003	−0.005	−0.006	−0.005	−0.001	0.002	0.001	0.004
Number of beds for 10,000 people in the city	−0.001	−0.01	−0.001	−0.011	0.003	0.006	0.003	0.010
Health index of the community	−0.441	−0.391	−0.421	−0.417	0.541 *	0.247	0.093	0.404
Service quality index of the community	−0.171	−0.199	−0.016	−0.212	0.125	0.134	0.178	0.219
Service quality index of the city	0.904 *	−0.401	0.398	−0.428	0.013	0.215	0.131	0.351
Intercept	7.307 ***	−1.581	6.513 ***	−1.685	9.371*	1.112	7.438 **	1.750

Note: ^†^ Reference levels in the regressions; Coef. for odds ratio; SE: standard error; * *p* < 0.05, ** *p* < 0.01, *** *p* < 0.001.

**Table 6:** Health service costs probability in different economic quintiles in China.

Economic Quantiles	Two-Week Outpatient		Inpatient	
	Total Cost/Yuan M (SD)	OOP/Yuan M (SD)	Total Cost/Yuan M (SD)	OOP/Yuan M (SD)
Poorest	462.40 (655.43)	414.63 (532.63)	6365.16 (5156.93)	4217.92 (4025.32)
Poorer	731.86 (643.27)	580.38 (482.36)	4353.10 (3476.60)	2641.95 (2230.21)
Middle	427.86 (482.23)	325.18 (347.20)	7039.63 (5739.53)	4540.65 (4436.11)
Richer	690.04 (682.13)	497.37 (4579.1)	7846.43 (5865.04)	4778.55 (5186.82)
Richest	572.48 (744.61)	418.21 (545.90)	8346.19 (6488.61)	5160.43 (5633.95)

Note: M for mean value; SE for standard error.

**Table 7:** CIs of two-week outpatient costs and inpatient costs among rural migrant workers with NCMS.

	CI	SE	*p-*Value	95% Confidence Interval	
				Lower Limit	Higher Limit
Total cost of outpatient	0.009	0.050	0.861	−0.005	0.133
OOP cost of outpatient	−0.026	0.060	0.658	−0.402	0.171
Total cost of inpatient	0.102	0.030	<0.01	0.031	0.149
OOP cost of inpatient	0.094	0.039	<0.05	0.007	0.119

**Table 8:** Concentration index decomposition of the total cost and OOP of two-week outpatient and inpatient among rural migrant workers with NCMS.

	Total Cost of Outpatient		OOP Cost of Outpatient		Total Cost of Inpatient		OOP Cost of Inpatient	
	dy/dx	Con/%	dy/dx	Con/%	dy/dx	Con/%	dy/dx	Con/%
36~50	−0.010	3.28	−0.023	11.16	0.094	16.72	0.097	14.06
50~64	0.019	9.14	0.027	12.46	0.224	−9.98	0.193	−6.99
Women	−0.056	−33.17	−0.041	−90.53	−0.397	121.41	−0.516	149.47
Live without spouse	−0.074	8.60	−0.065	11.47	−0.306	−20.37	−0.536	−29.01
Primary school	−0.001	0.21	0.024	−17.63	−0.046	−8.56	−0.102	−15.33
Middle school and above	0.008	−3.00	0.007	−11.62	−0.003	−0.47	0.073	8.64
Having technical certificate	−0.030	−3.81	0.009	4.91	−0.124	1.68	−0.107	1.18
Service stuff	0.006	−1.73	0.061	−63.44	−0.387	−28.22	−0.708	−41.93
Manufacturing and construction	0.039	4.20	0.061	17.21	−0.434	18.85	−0.693	24.48
Freelancer	0.018	5.98	0.059	10.20	−0.386	−9.74	−0.681	−13.97
Collective enterprises and institutions	−0.058	−0.49	−0.125	−14.42	−0.534	64.51	−0.556	34.59
Self-employed and freelance	−0.029	3.22	−0.100	16.14	−0.577	−74.35	−0.338	−35.40
Excessive labor	0.001	3.28	−0.046	10.14	−0.121	−17.90	−0.250	−29.94
Across the county/district	0.044	8.3	−0.013	−10.11	−0.726	−7.85	−0.969	−8.52
Poorer	0.025	87.59	0.031	159.31	0.052	−48.23	0.091	−40.05
Middle	−0.004	0.00	0.004	0.01	0.148	49.54	0.168	44.89
Richer	0.033	−56.57	0.043	−114.402	0.079	57.27	0.131	85.02
Richest	−0.008	37.04	−0.024	167.01	−0.008	−12.32	−0.010	−12.68
Having no injury insurance	−0.004	−0.55	0.065	17.07	0.567	−32.24	0.704	−32.55
number of friends 6~10	−0.004	0.98	−0.001	1.18	0.033	7.75	0.070	13.35
number of friends ≥ 11	0.004	−2.45	−0.006	15.17	0.012	5.81	0.047	18.60
Fair SAH	−0.009	−3.53	−0.001	−11.82	−0.025	−35.89	−0.021	−0.37
Poor SAH	0.006	8.44	0.008	40.51	0.108	−0.55	0.121	−32.53
No Smoking	0.007	13.46	0.049	10.44	0.353	−53.55	0.512	−86.69
No alcohol use	0.065	12.61	0.033	18.68	−0.187	18.95	−0.085	6.97
No regular exercise every month	−0.033	−3.54	−0.022	−10.04	0.027	−1.52	0.097	−4.43
Fair happiness	0.011	0.82	0.036	11.52	−0.002	0.46	0.081	−12.84
Happy	0.080	−9.53	0.113	−6.04	0.094	12.11	0.215	22.58
Proportion of ethnic minorities	0.017	12.78	0.016	19.58	−0.051	6.99	−0.049	5.55
Per capita in the community	0.010	−9.18	0.018	−49.57	0.034	19.25	0.038	10.30
Middle	0.016	6.77	0.008	13.75	0.048	5.09	−0.150	−12.88
West	0.013	7.77	0.008	18.74	−0.084	−9.21	−0.141	−12.51
Below Sub-provincial city	−0.054	−0.99	−0.144	−11.15	−0.047	−0.50	−0.333	−2.88
Number of medical institutions for 10,000 people in the community	−0.008	−2.07	−0.011	−12.59	−0.103	36.35	−0.141	40.56
Number of medical institutions for 10,000 people in the city	−0.017	−3.70	0.004	3.68	0.046	2.04	0.037	1.33
Number of beds for 10,000 people in the city	−0.051	13.53	−0.089	17.67	−0.127	−11.56	0.096	7.08
Health index of the community	−0.020	3.47	−0.027	19.61	0.435	18.57	0.211	7.30
Service quality index of the community	0.003	−14.39	0.003	−74.85	−0.048	6.43	−0.075	8.11
Service quality index of the city	−0.002	3.19	1.80 × 10^−4^	−1.32	0.033	−21.07	0.044	−24.97

Note: Con for Contributions.

**Table 9:** Horizontal index of the total cost and OOP of two-week outpatient and inpatient among rural migrant workers with NCMS.

	Total Cost of Outpatient		OOP Cost of Outpatient		Total Cost of Inpatient		OOP Cost of Inpatient	
	CI	Co	CI	Con/%	CI	Con/%	CI	Con/%
CI	0.009	100.00	−0.026	100	0.102	100.00	0.094	100.00
Need	−0.001	−15.84	0.010	−38.22	0.051	91.71	0.085	123.64
Economy	0.006	68.07	−0.055	211.9	0.026	46.27	0.053	77.19
Other	−0.002	−23.20	0.022	−86.49	−0.032	−57.07	−0.086	−124.82
residual	0.004	43.28	−0.003	12.81	0.011	19.09	0.017	23.98
HI	0.010		−0.036		0.051		0.009	

## Data Availability

All the data we used have been publicly released on the CLDS website: http://css.sysu.edu.cn/Data (accessed on 1 September 2021).

## Abbreviations

Hukou	Chinese household registration system
NCMS	New Rural Cooperative Medical Insurance
URBMI	urban residents’ basic medical insurance system
URRBMI	urban and rural residents’ basic medical insurance system
CLDS	China Labor-Force Dynamic Survey
SAH	self-assessed of health status
CI	concentration index
ICC	Intra-Class Correlation Coefficient
M	mean value
SD	standard deviation
ID	interquartile distance

## References

[1] Li D., Zhou Z., Shen C., Zhang J., Yang W., Nawaz R. (2020). Health Disparity between the Older Rural-to-Urban Migrant Workers and Their Rural Counterparts in China. Int. J. Environ. Res. Public Health.

[2] Zhong B., Liu T., Chan S., Jin D., Hu C., Dai J., Chiu H. (2018). Common mental health problems in rural-to-urban migrant workers in Shenzhen, China: Prevalence and risk factors. Epidemiol. Psychiatr. Sci..

[3] Song Y. (2014). What should economists know about the current Chinese hukou system?. China Econ. Rev..

[4] Liu X., Gao W., Yan H. (2014). Measuring and decomposing the inequality of maternal health services utilization in Western Rural China. BMC Health Serv. Res..

[5] Liu L., Ying I., Zhang J., Shi Y.H., Chang C. (2018). A study on health status and equity of health service utilization among floating population. Health Econ. Res..

[6] Hong M., Yue L. (2018). Health difference and influence factors of middle-aged and elderly migrant workers in China. Popul. Soc..

[7] Shao C., Meng X., Cui S., Wang J., Li C. (2016). Income-related health inequality of migrant workers in China and its decomposition: An analysis based on the 2012 China Labor-force Dynamics Survey data. J. Chin. Med. Assoc..

[8] Li D., Zhu L., Zhang J., Yang J. (2021). Decomposing Differences of Health Service Utilization among Chinese Rural Migrant Workers with New Cooperative Medical Scheme: A Comparative Study. Int. J. Environ. Res. Public Health.

[9] Andersen R.M. (1995). Revisiting the Behavioral Model and Access to Medical Care: Does It Matter?. J. Health Soc. Behav..

[10] Roh S., Burnette C.E., Lee K.H., Lee Y.S., Martin J.I., Lawler M.J. (2017). Predicting Help-Seeking Attitudes Toward Mental Health Services Among American Indian Older Adults: Is Andersen’s Behavioral Model a Good Fit?. J. Appl. Gerontol. Off. J. South Gerontol. Soc..

[11] Shao S., Wang M., Jin G., Zhao Y., Lu X., Du J. (2018). Analysis of health service utilization of migrants in Beijing using Anderson health service utilization model. BMC Health Serv. Res..

[12] Lu S., Li Y. (2018). Anderson’s the Behavioral Model of Health Services Use: Interpretation and Operationalization of Index System. Chin. Health Econ..

[13] Peng Y., Chang W., Zhou H., Hu H., Liang W. (2010). Factors associated with health-seeking behavior among migrant workers in Beijing, China. BMC Health Serv. Res..

[14] Zhao X., Ming D., Ma W. (2015). Utilization and cost of outpatient care and their influencing factors among middle and aged peasant-workers in China. J. Peking Univ. Health Sci..

[15] Jacobs J.A., Gersen K. (1998). Who Are the Overworked Americans?. Rev. Soc. Econ..

[16] Cha Y., Weeden K.A. (2014). Overwork and the Slow Convergence in the Gender Gap in Wages. Am. Sociol. Rev..

[17] Center for Health Statistics and Information The National Bureau of Statistics 2015 Survey Report on Rural-to-Urban Migrants. http://www.stats.gov.cn/tjsj/zxfb/201604/t20160428_1349713.html.

[18] Fabrigar L.R., Wegener D.T., Maccallum R.C., Strahan E.J. (1999). Evaluating the Use of Exploratory Factor Analysis in Psychological Research. Psychol. Methods.

[19] Bartlett M.S. (1992). Properties of Sufficiency and Statistical Tests. Proc. R. Soc. A Math. Phys. Eng. Sci..

[20] Cohen J. (1977). Statistical Power Analysis for the Behavioral Sciences.

[21] Johnston G., Wilkinson D. (2001). Increasingly inequitable distribution of general practitioners in Australia, 1986–1996. Aust. N. Z. J. Public Health.

[22] Wagstaff A., Doorslaer E.V. (2000). Measuring and testing for inequity in the deliveryof health care. J. Hum. Resour..

[23] Kakwani N., Wagstaff A., Doorslaer E.V. (1997). Socio-economic inequalities in health: Measurement, computation, and statistical inference. J. Econ..

[24] Van Doorslaer E., Masseria C., Koolman X. (2006). The OECD Health Equity Research Group. Inequalities in Access to Medical Care by Income in Developed Countries. Can. Med. Assoc. J..

[25] Yiengprugsawan V., Lim L.L., Carmichael G.A., Dear K.B., Sleigh A.C. (2010). Decomposing socioeconomic inequality for binary health outcomes: An improved estimation that does not vary by choice of reference group. BMC Res. Notes.

[26] O’Donnell O., Doorslaer E.V., Wagstaff A., Lindelow M. (2008). Analyzing Health Equity Using Household Survey Data: A Guide to Techniques and Their Implementation.

[27] Li D., Zhou Z., Si Y., Xu Y., Shen C., Wang Y., Wang X. (2018). Unequal distribution of health human resource in mainland China: What are the determinants from a comprehensive perspective?. Int. J. Equity Health.

[28] Skalkidou A. (2000). Parental family variables and likelihood of divorce. Soz. Präventivmedizin.

[29] Waldron I., Hughes M.E., Brooks T.L. (1996). Marriage protection and marriage selection—prospective evidence for reciprocal effects of marital status and health. Soc. Sci. Med..

[30] Hu X., Cook S., Salazar M.A. (2018). Internal migration and health in china. Lancet.

[31] Barsky A.J., Orav E.J., Bates D.W. (2005). Somatization increases medical utilization and costs independent of psychiatric and medical comorbidity. Arch. Gen. Psychiatry.

[32] Williams L.K., Andrianopoulos N., Cleland V., Crawford D., Ball K. (2013). Associations between education and personal income with body mass index among Australian women residing in disadvantaged neighborhoods. Am. J. Health Promot..

[33] Shavers V.L., Shankar S., Alberg A.J. (2002). Perceived access to health care and its influence on the prevalence of behavioral risks among urban African Americans. J. Natl. Med. Assoc..

